# Use of Contraceptive Methods Among Women in the General Population and Female Gynecologists in Spain: the ELEGIAN Survey

**DOI:** 10.1089/whr.2023.0048

**Published:** 2023-10-09

**Authors:** Josep Perelló-Capo, José C. Quílez-Conde, José Gutiérrez-Alés, Paloma Lobo-Abascal, Inmaculada Parra-Ribes, Ignacio Cristóbal-García, Mercedes Andeyro-García, Mercedes Herrero-Conde, Joan Rius-Tarruella, Joaquim Calaf-Alsina

**Affiliations:** ^1^ Department of Obstetrics and Gynecology, Hospital de la Santa Creu i Sant Pau, Barcelona, Spain.; ^2^ Departament of Pediatrics, Obstetrics and Gynecology, Preventive Medicine and Public Health, Universitat Autònoma de Barcelona, Barcelona, Spain.; ^3^ Department of Obstetrics and Gynecology, Hospital Universitario Basurto, Bilbao, Spain.; ^4^ Contraception and Sexual and Reproductive Health, Universidad de Alcalá (UAH), Madrid, Spain.; ^5^ Department of Obstetrics and Gynecology, Hospital Universitario Infanta Sofía, San Sebastián de los Reyes, Madrid, Spain.; ^6^ Biomedical and Health Sciences, Obstetrics and Gynecology, Universidad Europea, Madrid, Spain.; ^7^ Sueca Sexual and Reproductive Health Unit, La Ribera Department of Obstetrics and Gynecology, Conselleria de Sanitat Universal i Salut Pública, Valencia, Spain.; ^8^ Department of Obstetrics and Gynecology, Hospital Clínico San Carlos, Universidad Francisco de Vitoria, Madrid, Spain.; ^9^ Department of Obstetrics and Gynecology, Hospital General de Villalba, Madrid, Spain.; ^10^ Obstetrics and Gynecology Department, Faculty of Medicine, Universidad Alfonso X, Madrid, España.; ^11^ Department of Obstetrics and Gynecology, Gine4, HM Hospitals, Madrid, Spain.; ^12^ Medical Department, Bayer Hispania S.L., Barcelona, Spain.

**Keywords:** consumer behavior, contraception, contraceptive behavior, contraceptive prevalence survey, long-acting reversible contraception, patient preference

## Abstract

**Background:**

The contraceptive preferences of obstetricians and gynecologists (OB/GYNs) are thought to influence the contraceptive counseling they provide. The purpose of this study was to assess contraceptive preferences of OB/GYNs and women in the general population (WGP) in the current Spanish contraceptive scenario.

**Materials and Methods:**

Anonymous online survey of 100 OB/GYNs and 1,217 WGP aged 23–49 years.

**Results:**

WGP were younger (35.3 ± 7.3 vs. 37.9 ± 6.2 years, respectively) and less likely to have stable partners (64.7% vs. 84.0%) and children (49.1% vs. 62.0%) (all *p* < 0.05 vs. OB/GYNs). Seventy-nine percent versus 82%, respectively, used contraceptive methods, with condoms used most frequently by WGP (37% vs. 22% by OB/GYNs; *p* < 0.05) and pills by OB/GYNs (26% vs. 21% by WGP; *p* > 0.05). Intrauterine devices (IUDs) were more frequently used by OB/GYNs (20% vs. 5%; *p* < 0.05), especially the levonorgestrel-releasing intrauterine devices (LNG-IUDs) (18% vs. 2.6%; *p* < 0.05). The highest-rated methods were condoms among WGP and LNG-IUDs among OB/GYNs. Effectiveness was the most valued attribute of contraceptive methods for both. Reasons related to convenience were the main reason for choosing IUDs. OB/GYNs prescribed the contraceptive method in 40% of cases.

**Conclusions:**

Our study reveals differences between female OB/GYNs and WGP in contraceptive methods use and rating. The use of LNG-IUDs was much higher among OB/GYNs.

## Introduction

Health care professionals (HCPs) are thought to have a powerful influence on access to contraception, and choice and use of methods.^1^ However, knowledge of the contraceptive methods used by HCPs involved in contraceptive counseling and provision and how much their personal experiences and beliefs influence their advice is limited. A survey conducted in 2011 among 1,001 HCPs involved in contraceptive counseling (67.1% of whom were obstetricians and gynecologists [OB/GYNs] and 31.4% general practitioners [GPs]) from 10 countries, including Spain, showed that in seven of these countries, the most frequently used contraceptive method was levonorgestrel-releasing intrauterine devices (LNG-IUDs).^2^ Moreover, the personal use of LNG-IUDs by these HCPs was associated with a greater likelihood they would recommend them to women who had completed their childbearing.^2^ In a study conducted in the United States, Zigler et al. reported greater use of long-acting reversible contraceptives (LARCs) among OB/GYN residents versus the general population. These were mostly LNG-IUDs.^3^

The HABITS study, conducted in 2012, assessed the differences in contraceptive preferences between HCPs involved in contraceptive counseling and women in the general population (WGP) in Spain. A total of 300 female HCPs and 2,900 WGP participated in this study, in which condoms emerged as the preferred method in both populations (23% of female HCPs and 30% of WGP). Of interest, copper IUDs (Cu-IUDs) and the 52-mg LNG-IUD (the only LNG-IUD available at the time) were the second choice (13%) for HCPs and the fourth for WGP (7%).^4^ The impact of contraceptive preferences on prescription was not analyzed. Given the potential influence of the contraceptive preferences of OB/GYNs on the contraceptive counseling provided,^2^ knowing these preferences and how they change as the contraceptive scenario evolves may be important to reduce the rate of unintended pregnancies, which continues to be a burden in Spain.^5^ These potential influencers (the OB/GYNs) were poorly represented in the HABITS study (*n* = 50; 17% of all HCPs involved).^4^ Moreover, since the time of this study new contraceptive methods have become available. These include smaller LNG-IUDs with lower LNG doses (19 and 13.5 mg)—and a drospirenone-only oral contraceptive. Both IUDs are especially suitable for nulliparous women-only.^6^ Vaginal rings are now partially covered by the Spanish Healthcare system.

Updating and expanding this information allows the creation of a more realistic scenario, which further confirms disparities in access to and availability of certain contraceptive methods and analyzes the reasons for this (information, financial, *etc*.) and ways to ensure the provision of more equitable health care. With this aim, we have conducted a study with a greater sample of female OB/GYNs (*n* = 100) to gain updated insights into preferences concerning the use of contraceptive methods in WGP and female OB/GYNs. Secondary objectives included investigating how they rated the different options and the attributes of the contraceptives leading to their choice. We also assessed the role of OB/GYNs as contraceptive prescribers.

## Materials and Methods

### Design and participants

The ELEGIAN survey was an anonymous online survey conducted in Spain between November 23, 2021 and January 24, 2022 among 100 female OB/GYNs actively involved in contraceptive counseling and 1,217 WGP. This survey addressed several topics related to sexuality, contraception, and menstruation. Only topics related to contraception are reported here.

As per the HABITS study, to be eligible, all the women had to be aged 23–49 years and live in Spain. OB/GYNs needed to have at least 3 years of professional experience (including 3 years of postgraduate training to acquire competence in contraception, in accordance with Spanish legislation^7^) and be actively involved in contraceptive counseling. This meant that the lower age limit for OB/GYNs was 26 years. This was also a requirement in the international study conducted by Gemzell-Danielsson et al.^2^ We chose 23 years as the lower age limit for WGP to gain insight into the contraceptive preferences of younger women. Exclusion criteria were being menopausal and, for WGP, being an OB/GYN. The survey was conducted by Amber Marketing Research S. L. and was funded by Bayer Hispania S.L. The sponsor had no contact with or influence on the participants. Given the anonymous nature of the survey, no patient consent or ethical approval was needed under Spanish Health laws.

### Sampling strategy

WGP were selected by the consultancy company using their volunteer online panel, which comprises 13,125 female internet users from the general population. Of these, 8,251 were aged 23–49 years. Menopausal status was assessed in the first questions of the survey. OB/GYNs were selected using the OB/GYN contact list owned by Amber Marketing Research S. L., which includes HCPs in both the public and private sectors. This list was added by contacting OB/GYNs through their places of work. OB/GYNs were blinded to the study sponsor. Participation in the survey was voluntary for WGP, whereas OB/GYNs received remuneration. Payments were made subject to the provisions of the Code of Practice for the Pharmaceutical Industry (Farmaindustria).

### Sample size

As per the HABITS study, stratified random cluster sampling was used initially to obtain a sample representative of the general population. According to 2020 data from the Instituto Nacional de Estadística (National Statistics Institute),^8^ the number of women aged 23–49 years living in Spain was 8,717,465, of whom 1,743,493 (20%) were aged 23–29 years, 3,051,113 (35%) 30–39 years and 3,992,859 (45%) 40–49 years. Data from the Spanish Health Ministry show that in 2018 there were 3,303 OB/GYNs in Spain.^9^ The sample size was determined to be 1,200 women (sample error of ±2.8%), a figure similar to that of the regular surveys on contraceptive use undertaken by the Spanish Society of Contraception,^10^ when only the age group included in our study is considered. A sample size of 100 was established for female OB/GYNs (sampling error of ±9.6%).

The sampling error was calculated using an infinite WGP population (*n* = Z^2^ × p × q/e^2^) and a finite female OB/GYN population (*n* = Z^2^ × p × q × N/e^2^ (N − 1) + Z^2^ × p × q) as the basis, with a 95.5% confidence level and *p* = *q* = 0.5. A total of 400 women were included in each age group (23–29, 30–39, and 40–49 years old) so as to be able to detect differences according to age. A minimum of 30 interviews per each Spanish autonomous community were conducted to detect differences among them. Data were weighted by autonomous community and age according to 2020 data.^8^

### Data collection

Computer-assisted web interviewing was used for data collection through a semi-structured questionnaire. The questionnaire was based on the one used in the HABITS study with minor modifications aimed at capturing new developments in the contraceptive landscape. The questionnaire was shared with the investigators' panel in a virtual meeting and approved unanimously before being launched. The panel is composed by members of the steering committee of the Spanish societies of contraception and participates in the regular surveys on contraception.

The questionnaire consisted of two parts, the first of which gathered information on sociodemographics, relationship status, and parity. The second part focused on the specific topics being investigated. The section devoted to contraceptive preferences and use consisted of 12 multiple-choice questions. The contraceptive method reported was the one in use at the time of the study. Condoms were considered only as a single method (*i.e*., not as part of a dual contraceptive method). The rating of contraceptive methods was conducted using a list of methods that included short- and long-term reversible methods and condoms. No definitive methods were included. Participants received the link to the questionnaire by e-mail. Fifteen percent of the interviews underwent quality control to guarantee the quality of the results.

The general term “contraceptive pills” was used for WGP given the difficulty of these women to differentiate between combined oral contraceptives (COCs) and the progestin-only minipills. OB/GYNs were asked to select one or the other.

### Statistical analysis

The data were described using distribution of frequencies for categorical variables. These were compared using the chi-square test. Frequencies and mean scores (plus standard deviation when appropriate) were calculated to show assessment results using Likert scales. Mean scores were compared using Student's *t*-test. The data are presented by age group in WGP when considered relevant. Age differences are not presented for OB/GYNs, given the small sample size. A two-sided value of *p* < 0.05 was considered significant. The statistical analysis was performed by Amber Marketing Research S. L. with supervision by the first author. This analysis included all the sub analyzes deemed relevant to interpret the results as requested by the authors. The statistical package used was Barbwin 7.5.

## Results

### Participants

Of 4,478 and 215 e-mails sent to WGP and female OB/GYNs, respectively, 1,217 WGP and 100 OB/GYNs completed the survey. They were aged 35.3 ± 7.3 and 37.9 ± 6.2 years, respectively (*p* < 0.05). The OB/GYNs' average length of professional experience was 12.1 years, with 49% of them having >10 years of experience. WGP were less likely to have stable partners (64.7% vs. 84.0%) and children (49.1 vs. 62.0) (both *p* < 0.05 vs. OB/GYNs). The length of the current relationship and the number of sexual partners were similar in both populations (Table 1).

**Table 1.** Baseline Characteristics of Participants

	WGP (***n*** = 1,217)	Female OB/GYNs (***n*** = 100)	***p***
Age, years, mean (SD)	35.3 (7.3)	37.9 (6.2)	<0.05
23–30 years, *n* (%)	401 (32.9)	14 (14.0)	<0.05
31–39 years, *n* (%)	406 (33.4)	44 (44.0)	<0.05
40–49 years, *n* (%)	410 (33.7)	42 (42.0)	<0.05
Stable partner, yes, *n* (%)	982 (80.6)	91 (91.0)	—
Living together	788 (64.7)	84 (84.0)	<0.05
Not living together	194 (15.9)	7 (7.0)	<0.05
Length of relationship, years, mean (SD)			
Living together	11 (7.3)	13 (7.5)	**—**
Not living together	4 (4.6)	1 (1.0)	**—**
Number of sexual partners in the last year^a^, mean (SD)	1.3 (1.8)	1.4 (1.4)	**—**
Children, yes, *n* (%)	598 (49.1)	62 (62.0)	<0.05
Number of dependent children^b^, mean (SD)	1.6 (0.7)	2.0 (0.7)	<0.05

Statistical differences were analyzed using the chi-square test for categorical and Student's *t*-test for continuous variables.

^a^ Only in women without a stable partner.

^b^ Only in women with children.

OB/GYNs, obstetricians and gynecologists; SD, standard deviation; WGP, women in the general population.

### Contraceptive use

The percentage of women using any contraceptive method at all at the time of the survey was similar in both populations (79% of WGP [*n* = 959] and 82% of OB/GYNs [*n* = 81]; *p* > 0.05). Condoms were the most widely used contraceptive method among WGP (47% vs. 27% among OB/GYNs when only women using a contraceptive method were considered, *p* < 0.05; 37% vs. 22% among OB/GYNs when the overall population was considered). Contraceptive pills were the most widely used contraceptive method for OB/GYNs (32% vs. 27% for WGP, *p* > 0.05 and 26% vs. 21% in the overall population). IUDs were the third most frequently used method, although more so by OB/GYNs than by WGP (25% vs. 6%, *p* < 0.05 and 20% vs. 5% in the overall population). Among OB/GYNs using IUDs (20%), 90% used LNG-IUDs (18% of OB/GYNs).

**FIG. 1.**
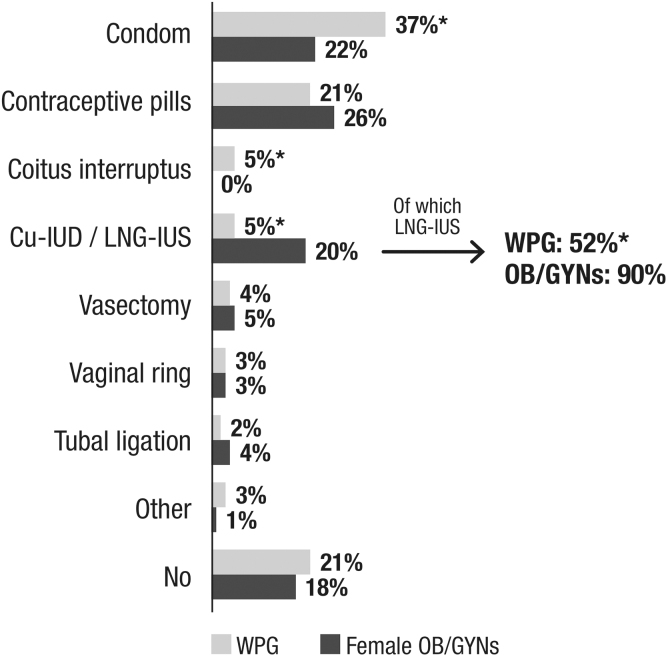
Use of contraceptive methods among WGP and female OB/GYNs. Q: Which contraceptive method are you currently using? (only one answer) Other: WGP: 1% subdermal implant, 1% contraceptive patch, 1% intradermal injection/OB/GYNs: 1% contraceptive patch, 1% not specified. *p < 0.05 Statistical differences were analyzed using the chi-square test. Cu-IUD, copper intrauterine device; LNG-IUD, levonorgestrel-releasing intrauterine device; OB/GYNs, obstetricians and gynecologists; WGP, women in the general population.

Among WGP using any IUD (5%), 52% used LNG-IUDs (2.6%; *p* < 0.05 vs. OB/GYNs) (Fig. 1). Among WGP, the use of condoms and contraceptive pills decreased with age (from 42% and 29% at the ages of 23–30 years, respectively, to 34% and 14% at the age of 40–49 years; *p* < 0.05). The use of IUDs barely increased with age (from 4% at the age of 23–30 to 5% at the ages of 40–49 years, *p* > 0.05).

### Rating of contraceptive methods

**FIG. 2.**
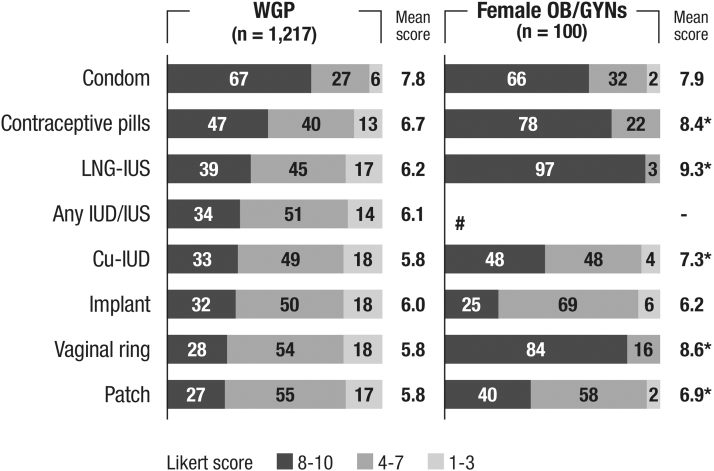
Rating of contraceptive methods by WGP and female OB/GYNs (overall population). Q: Overall, how do you rate the following contraceptive methods? (closed list of methods) (1 [Very negatively] – 10 [Very positively] Likert scale). ^#^Two gynecologists did not specify the type of IUD. *p < 0.05 Statistical differences were analyzed using the Student's *t*-test.

The highest rated contraceptive methods were condoms (mean score 7.8), contraceptive pills (6.7), and LNG-IUDs (6.2) for WGP and LNG-IUDs (9.3), vaginal rings (8.6) and contraceptive pills (8.4) for OB/GYNs. The differences in ratings between WGP and OB/GYNs were statistically significant except for subdermal implants and condoms (Fig. 2).

### Reasons for choosing a contraceptive method

**FIG. 3.**
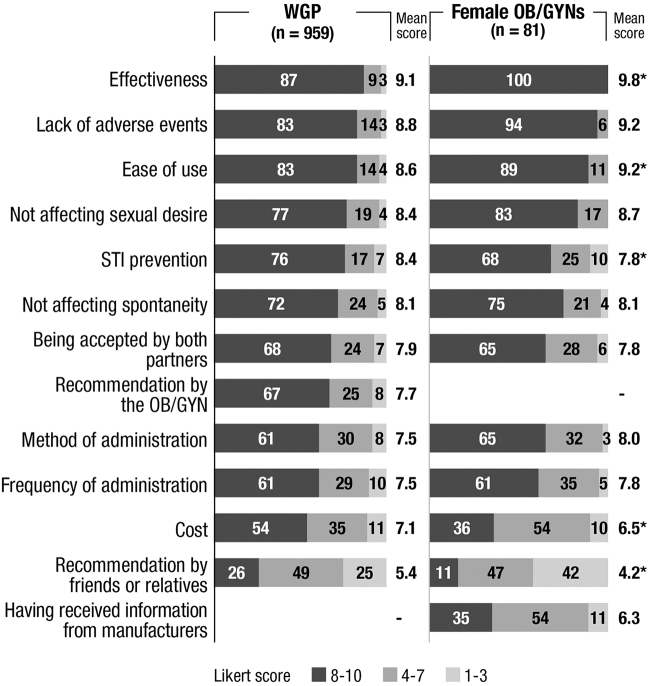
Most rated attributes when choosing a contraceptive method (only WGP and OB/GYNs using any method). Q: Please indicate the importance of the following characteristics when choosing a contraceptive method (1 [Not important at all] – 10 [Very important] Likert scale). *p < 0.05 Statistical differences were analyzed using the Student's *t*-test. STI, sexually transmitted infections.

Among women using contraceptive methods, the most highly rated attributes for the selection of a given contraceptive method were similar in both populations: effectiveness was rated most highly, especially in OB/GYNs (mean score of 9.8 vs. 9.1 in WGP; *p* < 0.05), followed by safety (9.2 vs. 8.8, respectively; *p* > 0.05) and ease of use, especially in OB/GYNs (9.2 vs. 8.6, respectively; *p* < 0.05). Recommendation by friends/relatives had the least influence on the decision, although it was more important for WGP (5.4 vs. 4.2 in OB/GYNs; *p* < 0.05). Price also was more important for WGP (7.1 vs. 6.5, respectively) (Fig. 3).

**FIG. 4.**
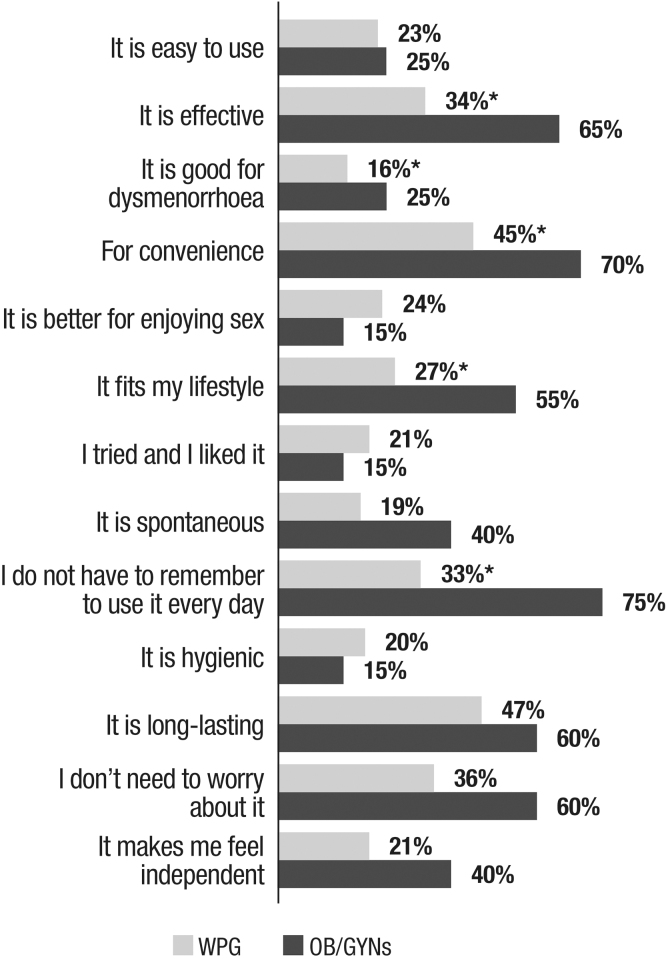
Reasons for using Cu-IUD/LNG-IUD among users (*n* = 55 for WGP and *n* = 20 for female OB/GYNs). Q: Which are the main reasons for choosing a Cu-IUD/LNG-IUD as your contraceptive? Multiple answers. Multiple choices. Only reasons reported by at least 20% of WGP or OB/GYNs are shown. *p < 0.05 Statistical differences were analyzed using the chi-square test.

The main reasons for choosing IUDs among OB/GYNs (*n* = 20; 20%) were not having to remember to use it every day (75%) and convenience (70%), followed by effectiveness (65%), all *p* < 0.05 versus WGP. Among WGP (*n* = 55; 5%), the reasons were more diverse, with length of use (47%), convenience (45%). and not having to worry about it (36%) rating highest. Differences between WGP and OB/GYNs in choosing IUDs were related to effectiveness, lifestyle, and convenience (Fig. 4).

### Role of OB/GYNs in the selection of contraceptive methods

Sixty-six percent of WGP reported having consulted an HCP (GP or OB/GYN) when starting the contraceptive method used at the time of the study (possible answers: yes/no). This percentage decreased with age: from 70% of women aged 23–30 years to 63% of those aged 40–49 years (*p* < 0.05). Consultations were more frequent for IUDs (93% of users), contraceptive pills (92%), and vaginal rings (90%).

OB/GYNs considered that 48% of contraceptive consultations were about contraceptive pills (specifically, COCs), 25% about Cu-IUDs or LNG-IUDs (18% and 7%, respectively), 11% about subdermal implants, and 10% about vaginal rings. Information about condoms accounted for 2% of consultations.

For 40% of WGP using contraceptive methods (*n* = 959), these had been prescribed by their OB/GYN. The woman's own choice accounted for 34% of cases. The role of the OB/GYN barely changed with age in WGP (from 43% at age 23–30 years to 37% and 41% at ages 31–39 and 40–49 years, respectively, *p* > 0.05). Conversely, the woman's own choice increased significantly (from 27% to 39% in the youngest and oldest age segments; *p* < 0.05). The role of the OB/GYN was more prominent for users of IUDs (71%) and contraceptive pills (62%), and less for condom users (25%). A woman's own choice was more prominent for condoms and less so for users of IUDs and contraceptive pills (52%, 13% and 8%, respectively).

## Discussion

The results of our study show significant differences between OB/GYNs and WGP when it comes to contraceptive use, appraisal, and drivers for choosing a contraceptive method. WGP opted for condoms as the preferred choice, followed by contraceptive pills and, in a lower percentage, IUDs. Contraceptive pills were the contraceptive method most frequently used by OB/GYNs, followed by condoms. The most highly rated contraceptives by OB/GYNs were LNG-IUDs, whereas condoms were for WGP. Effectiveness was the attribute most highly rated by WGP and OB/GYNs when choosing a contraceptive method. The method used by WGP was prescribed by an OB/GYN in 40% of cases, with this percentage increasing in the case of IUDs (71%).

The preferred methods for OB/GYNs were contraceptive pills (32%), condoms (27%), and IUDs (25%), which contrasts with the value placed on the different contraceptive options, which was higher for IUDs, with a mean score of 9.3, vaginal rings (8.6), and contraceptive pills (8.4). The use of IUDs also contrasts with the findings of the multinational survey conducted by Gemzell-Danielsson et al.,^2^ where 39.9% of OB/GYNs or their partners used IUDs (33.5% LNG-IUDs). Differential characteristics of both populations such as mean age (lower in our study), relationship status, and reproductive perspective, among other factors, may also affect the results. The use of IUDs by HCPs (17% OB/GYNs) in the HABITS study was also low (13%).^4^ Reasons for this should be investigated.

As reported in this study,^4^ we also found greater use of IUDs among OB/GYNs compared with WGP (20% vs. 5%), this being especially true for LNG-IUDs (18% vs. 2.6%). This finding is also consistent with the results of a survey conducted in the United States where female family planning providers aged 25–44 years used LARC more frequently than WGP of the same age (41.7% vs. 12.1%, *p* < 0.001) even when adjusting for race/ethnicity or educational level.^11^ Another survey conducted in the United States also found higher use of LARC in OB/GYN residents versus WGP (49% vs. 12%). Those affiliated with special contraceptive training programs were more likely to use LARC.^3^

The high use of condoms by WGP as the single contraceptive method (37%) reflects the great value placed on this contraceptive method based on their rating (mean score 7.8). In fact, condoms are so far the contraceptive method that is most frequently used by women of childbearing potential of all ages in Spain (31.3%), followed at some distance by oral contraception (18.5%; used by 21% of women in our study).^10^ Similarly, in the HABITS study, condoms were used by 30% of WGP and contraceptive pills by 17%.^4^ The greater use of condoms over oral contraception in Spain differs significantly from the contraceptive habits of other European countries, where oral contraception is more widespread.^12^ The weight of HCP's contraceptive counseling in this scenario is unknown.

In our study, the use of IUDs by WGP was low (6% among contraceptive users), despite being very positively evaluated by this population (third in the contraceptive rating). The use of other LARCs such as subdermal implants was even lower (1%), which agrees with the findings of the 2020 National contraceptive survey conducted in Spain.^10^ These findings support the existence of barriers to using these methods such as fears and misconceptions, but there are also economic barriers as their coverage by the National Healthcare System is not homogeneous across the Spanish autonomous communities. The use of Cu-IUDs/LNG-IUDs in Spain has barely increased in recent years according to regular surveys conducted in our country: from 3.6% and 2.5% of women using Cu-IUDs or LNG-IUDs in 2014^13^ to 2.9% and 3.8%, respectively, in 2022.^10^

Effectiveness was the main reason for choosing the contraceptive method in both populations, followed by safety. This finding is consistent with the results of the Kopp Kallner’ Nationwide Survey, where the most important characteristic when choosing a contraceptive method was its effectiveness (64.2%), followed by safety (61%).^14^ The importance given by WGP to contraceptive effectiveness in our study is especially relevant, as this attribute is thought to influence their contraceptive choices. Of interest, their first choices are condoms first and contraceptive pills next, both known to be highly dependent on user adherence.^15^ This is notable because many unintended pregnancies result from inconsistent or incorrect use of user-dependent methods.^16^ Reasons for choosing a contraceptive method vary with personal circumstances.^2^ This aspect was not analyzed in our study.

As in the international study conducted by Gemzell-Danielsson et al.,^2^ effectiveness and reasons related to convenience were the main drivers to choose IUDs by OB/GYNs. Reasons for choosing IUDs were, however, quite heterogeneous among WGP. Moreover, WGP did not associate what they considered to be the most important attributes of a contraceptive method (*i.e*., effectiveness, safety, and ease of use) with their main reasons for choosing IUDs. Instead, as in the HABITS study,^4^ reasons related to convenience were the main ones, with effectiveness following at a distance.

Consistent with the last national survey on contraception conducted in our country (2020),^10^ we also found that 66% of WGP had consulted a GP or OB/GYN before starting to use a contraceptive method (40% were OB/GYNs). This percentage was much more significant among WGP using IUDs (93%) or contraceptive pills (92%), probably because both contraceptive methods require an assessment of user eligibility and a prescription. OB/GYNs reported that contraceptive pills accounted for most consultations on contraception (48%), followed at a distance by IUDs (25%). This makes sense given the much higher frequency of use of contraceptive pills and is consistent with the OB/GYNs' prescribing role found in our study.

Limitations of our study include the lack of representativeness of our sample with regard to the overall Spanish population of WGP and the reduced size of the OB/GYN sample. The differential ways of selecting participants are also likely to create a bias. OB/GYNs were required to have 3 years of professional experience to ensure proper contraceptive training; however, this resulted in the proportion of OB/GYNs aged 23–30 years being much lower compared with that of WGP (14.0% and 32.9%, respectively). This may bias the results, as younger women are less frequently advised and offered information about LARC and more frequently use condoms or pills.^10^ Moreover, women at this age are less likely to get pregnant, at least in Spain, where the mean age of women at first birth is nearly 32 years.^17^

In fact, more OB/GYNs had children than WGP. This is likely to have an impact on the method used. Age-subgroup analysis should be conducted to better understand whether there are differences in access to highly effective methods. LNG-IUDs were clustered together despite the important differences between the different types because WGP might not differentiate between them. At present, there are no data on the use of the different types of LNG-IUDs in our setting. Differences in access to Cu-IUDs/LNG-IUDs because of differential financing of these methods across administrative regions in Spain should also be considered when interpreting these results. The strengths of our study include the greater number of OB/GYNs (*n* = 100) versus the HABITS study (*n* = 51).^4^ The OB/GYNs in our study were only female and were, therefore, a more homogeneous population. There were no missing answers. To our knowledge, our study is also the first to show how OB/GYNs rate the different contraceptive options.

## Conclusions

Female OB/GYNs and WGP show different contraceptive use, appraisal, and drives for choosing the contraceptive method. Contraceptive pills were the most frequently used method among OB/GYNs, whereas condoms among WGP. LNG-IUDs were more frequently used by OB/GYNs. Efforts are still needed both in OB/GYNs and WGP to foster the use of highly effective contraceptive methods.

## Abbreviations Used

COC: combined oral contraceptive
Cu-IUD: copper intrauterine device
GP: general practitioner
HCP: health care professional
LARC: long-acting reversible contraceptive
LNG-IUD: levonorgestrel intrauterine device
OB/GYN: obstetricians and gynecologist
SD: standard deviation
STI: sexually transmitted infections
WGP: women in the general population
